# New Trends in Statistical Physics of Complex Systems

**DOI:** 10.3390/e20120906

**Published:** 2018-11-27

**Authors:** Antonio M. Scarfone

**Affiliations:** Istituto dei Sistemi Complessi, Consiglio Nazionale delle Ricerche (ISC-CNR), c/o DISAT, Politecnico di Torino, Corso Duca degli Abruzzi 24, I-10129 Torino, Italy; antonio.scarfone@polito.it

**Keywords:** generalized statistical mechanics, information theory, anomalous diffusion, stochastic processes, collective phenomena, disordered systems

A challenging frontier in physics concerns the study of complex and disordered systems. They are characterized by an elevated level of interconnection and interaction between the parts, with a richer global dynamic, which gives rise to the collective emerging behaviour of the entire system that are no longer recognized in the properties of the single individual entities. In this scenario, the methods of statistical physics, in understanding the properties of complex systems, have proved to be very promising.

In fact, statistical physics is so general that it still holds, in a much wider context, than that, on which the original theory was developed. Consequently, despite its largely recognized success in physics, considerable efforts have been made to extend the formalism of statistical physics, beyond its original application. In this way an increasing amount of physical and physical-like systems are now well-studied by using the standard tools of this theory.

Thus, in the last decades, we assisted in an intense research activity that has modified our comprehension of statistical physics, extending and renewing its applicability, considerably. Important developments, relating equilibrium and nonequilibrium statistical mechanics, kinetic theory, information theory, and others, have produced a new understanding of the properties of complex systems that requires, in many cases, the extension of the statistical physics theory, beyond its standard formalism.

In this volume, we have selected and invited a limited number of papers on several topical trends in the statistical physics of complex systems, based on the talks presented during the international conference SigmaPhi2017 held in Corfu, from the 10th until the 14th of July 2017 (http://sigmaphisrv.polito.it/), which gathered together, more than two hundred and fifty international scientists, working in all areas of physics and interdisciplinary applications in non-physical systems.

This special issue collects twelve regular research articles, which underwent the standard rigorous editorial process of the Entropy journal.

The first three articles [1,2,3] deal with non-extensive statistical mechanics and power-law distributions.

The paper “Equilibrium States in Two-Temperature Systems” by E.M.F. Curado and F.D. Nobre, proposed a non-linear (power-like) Fokker-Planck equation, describing a kinetic process governing the evolution toward the equilibrium of a system, whose underlying dynamics was characterized by two different diffusional mechanisms. The diffusion term in the kinetic equation is related to the sum of the two entropic forms, each of them associated to a given diffusion process. The corresponding diffusion coefficients introduce two distinct characteristic temperatures of the system, a situation that usually appears in nonequilibrium statistical mechanics. A physical application to type-II superconducting vortices was discussed as an illustrative example.

In the paper “Information-Length Scaling in a Generalized One-Dimensional Lloyd’s Model” by J.A. Méndez-Bermúdez and R. Aguilar-Sánchez, a numerical study of the generalized one-dimensional Lloyd’s model was presented. This model described a class of disordered systems characterized by a random variable whose density distribution function exhibits an asymptotically-slow decaying tail. It was, in a sense, related to several disordered models that have been studied in the literature, like the bended random matrix model, the kicked-rotator model, the multiplex and multilayer random networks, and others, so that the result obtained in this paper could have a wide spectrum of applicability.

The paper “Oscillations in Multiparticle Production Processes” by G. Wilk and Z. Włodarczyk, dealt with the study of the power-law and quasi-power-law distributions, characterized by log-periodic oscillations that are ubiquitously observed in many different branches of science. Based on the nonextensive statistical mechanics, the authors presented a study of the available experimental data from the Large Hadron Collider (LHC) experiments, obtained from the multiparticle production processes, at high energies. The analysis concerned: (i) The log-periodic oscillations pattern decorating the power-like Tsallis distributions of the large transverse momenta spectra, and (ii) the oscillations of some coefficients in the recurrence relation defining the multiplicity of distributions.

The information theory is covered by the next three articles [4,5,6].

In the paper “Minimising the Kullback-Leibler Divergence for Model Selection in Distributed Nonlinear Systems” by O.M. Cliff, M. Prokopenko, and R. Fitch, the authors discussed, in the context of a nonlinear dynamical system, a possible decomposition of the Kullback-Leibler divergence, a fundamental quantity in information theory, in two information-measures, namely collective transfer entropy and stochastic interaction. This result has been derived by using rigorous methods, based on differential topology and, as highlighted in the paper, the approach proposed has potential applications beyond the study of a complex system theory, becoming relevant in a variety of contexts of artificial intelligence, like in machine learning.

The next paper “Conformal Flattening for Deformed Information Geometries on the Probability Simplex” by A. Ohara, dealt with the study of two important notions that play a relevant role in information geometry in characterizing statistical models with generalized exponential functions. The first one, the dual flatness, produced fruitful geometrical structures like the existence of canonical coordinate systems, a pair of conjugate potential functions and assures the existence of a canonical divergence. The second one, was concerned with the invariance of a geometric structure, a crucially valuable concept in developing mathematical statistics, which holds only when the statistical manifold has a special triplet given by the Riemannian metric and a pair of mutually dual-affine connections.

Always within the information geometry, the paper “The Volume of Two-Qubit States by Information Geometry” by M. Rexiti, D. Felice and S. Mancini, studied the volume of the set of two-qubits states with maximally disordered subsystems, by considering their phase space representation, in terms of probability distribution functions and by applying the classical Fisher information metric. The results obtained were compared with those derived by using two different versions of the quantum Fisher metric—the Helstrom and the Wigner–Yanase-like metrics. Although the absolute values of volumes of separable and entangled states differ in the two approaches, it was shown that their ratios are comparable, supporting the conclusion that classical Fisher information is able to capture the features of the volume of quantum states.

The last topic collects six articles and deals with collective phenomena, respectively, in condensed matter [7,8,9,10] and nuclear matter [11,12].

In the paper “Collective Motion of Repulsive Brownian Particles in Single-File Diffusion with and without Overtaking” by T. Ooshida, S. Goto and M. Otsuki, a system of repulsive Brownian particles confined in a (quasi-)one-dimensional channel, whose sub-diffusive behavior is known as the single-file diffusion, was studied. Collective dynamics was illustrated by the calculations of the two-particle displacement correlation of the system. It was shown both numerically and analytically that the overtaking processes only destroy the short-range correlations, leaving the long-range correlations nearly intact. Numerical solutions to the Langevin equation, with a large but finite interaction potential, were studied to clarify the effect of overtaking, while, when particles are allowed to overtake each other and, thereby, escape from the quasi-1D cage as a rare event, the effect of a non-zero overtaking rate on the displacement correlation was derived analytically.

In the paper “Strong- and Weak-Universal Critical Behaviour of a Mixed-Spin Ising Model with Triplet Interactions on the Union Jack (Centered Square) Lattice” by J. Strečka, the mixed spin-1/2 and spin-S Ising model, with a triplet interaction on the centered square lattice, was studied, by establishing a rigorous mapping correspondence with the symmetric eight-vertex model. This exact mapping equivalence was showed by employing two independent mechanisms—by exploiting the graph-theoretical formulation and by using the spin representation of the zero-field eight-vertex model. Then, it was shown that the critical exponents of the mixed spin-1/2 and spin-S Ising model, with a triplet interaction, fundamentally depend on the interaction anisotropy, as well as on the spin parity. This was in contrast to the universality conjecture, which states that the critical behavior of very different models may be characterized by the same set of critical exponents.

In the paper “Anomalous Statistics of Bose-Einstein Condensate in an Interacting Gas: An Effect of the Trap’s Form and Boundary Conditions in the Thermodynamic Limit” by S. Tarasov, V. Kocharovsky, and V. Kocharovsky, the authors studied a mesoscopic system formed by a finite number of trapped particles in a three-dimensional rectangular box, with Dirichlet boundary conditions, in the mean-field Bogoliubov and Thomas-Fermi approximations. It was shown that this model yields the non-Gaussian condensate occupation statistics which is different from the non-Gaussian ideal-gas BEC statistics but shares with it, a similar dependence on the trap form and on the boundary conditions. Such a dependence does not vanish in the thermodynamic limit with the increase of the interparticle interaction, the number of trapped particles, and the volume of the system.

In the paper “Study on Bifurcation and Dual Solutions in Natural Convection in a Horizontal Annulus with Rotating Inner Cylinder Using Thermal Immersed Boundary-Lattice Boltzmann Method” by Y. Wei, Z. Wang, Y. Qian, and W. Guo, the mechanism of the rotation effect on bifurcation and on dual solutions, in a natural convection of a horizontal annulus, was studied, numerically. The mechanism of the rotation effect was quantified by linear speed of rotational inner cylinder and it was presented and analyzed by the streamlines and isotherms at different dimensionless linear speeds. The obtained results manifested the existence of three convection patterns which affect the heat transfer in different ways, where the linear speed determines the proportion of each convection.

The paper “Mathematical Realization of Entropy through Neutron Slowing Down” by B. Ganapol, D. Mostacci, and V. Molinari, deals with the study of a classical problem in the neutron transport theory. It was shown that the slowing down equation for elastic scattering of neutrons in an infinite homogeneous medium can be solved analytically. These solutions characterized the evolution of disorder associated with neutron–nucleus collisions. Starting from the monoenergetic neutrons configuration, that represents a complete ordering system, the subsequent scattering creates disorder by uniformly redistributing the neutron energy and the recoil energy transfer to field particles showing, in this way, an increasing entropy with increasing lethargy.

Finally, in the paper “Energy from Negentropy of Non-Cahotic Systems” by P. Quarati, A.M. Scarfone, and G. Kaniadakis the role of positive and negative contributions of entropy and free energy were explored, with their constraints, during a transition of a system, from a non-equilibrium to an equilibrium chaotic state. The amount of negentropy of a non-chaotic system is a source of energy that can be transferred to an internal or inserted subsystem. The subsystem increases its energy and can perform processes that otherwise would not happen like, for instance, the nuclear fusion of the inserted deuterons in a liquid metal matrix, among many others. A few evaluations concerning the non-ideal molecular gas, warm dense matter, and the nuclear matter were reported.
